# Leflunomide-Induced Hidradenitis Suppurativa

**DOI:** 10.1155/2020/3549491

**Published:** 2020-02-18

**Authors:** Achraf Machan, Hasna Azendour, Hamza Toufik, Lahsen Achemlal, Mohammed Boui, Naoufal Hjira

**Affiliations:** ^1^Department of Dermatology, Mohammed V Military Hospital, Mohammed V University of Rabat, Rabat, Morocco; ^2^Department of Dermatology, CHU Ibn Sina, Mohammed V University of Rabat, Rabat, Morocco; ^3^Rheumatology, Mohammed V Military Hospital, Mohammed V University of Rabat, Rabat, Morocco

## Abstract

Hidradenitis suppurativa is an inflammatory disease of the pilosebaceous unit with a chronic intermittent course and a devastating effect on quality of life. Rare reports of drug-induced hidradenitis suppurativa exist. We report on 2 women on follow-up for rheumatoid arthritis, who presented hidradenitis suppurativa after different periods of treatment with leflunomide and who improved few weeks after discontinuation of the medication.

## 1. Introduction

Hidradenitis suppurativa (HS) is an inflammatory disease of the pilosebaceous unit with a chronic intermittent course and a devastating effect on quality of life. Association with obesity, smoking, family history of the disease, and other inflammatory conditions is well identified [1]. Rare reports of drug-induced HS exist. We report 2 cases of HS induced by leflunomide. To the best of our knowledge, these are the first 2 cases of leflunomide-induced HS reported in the literature.

## 2. Case Presentation

### 2.1. Case 1

The patient was a 66-year-old woman, nonsmoker, being overweight with a BMI of 28 kg/m^2^, with no relevant family history of HS, with a 14-year personal history of rheumatoid arthritis, treated initially with methotrexate and glucocorticoids, actually at 5 mg/day of prednisone PO.

The patient presented 7 months ago an intolerance to methotrexate, which prompted the withdrawal of the latter and its substitution with leflunomide 20 mg/day.

**Figure 1:**
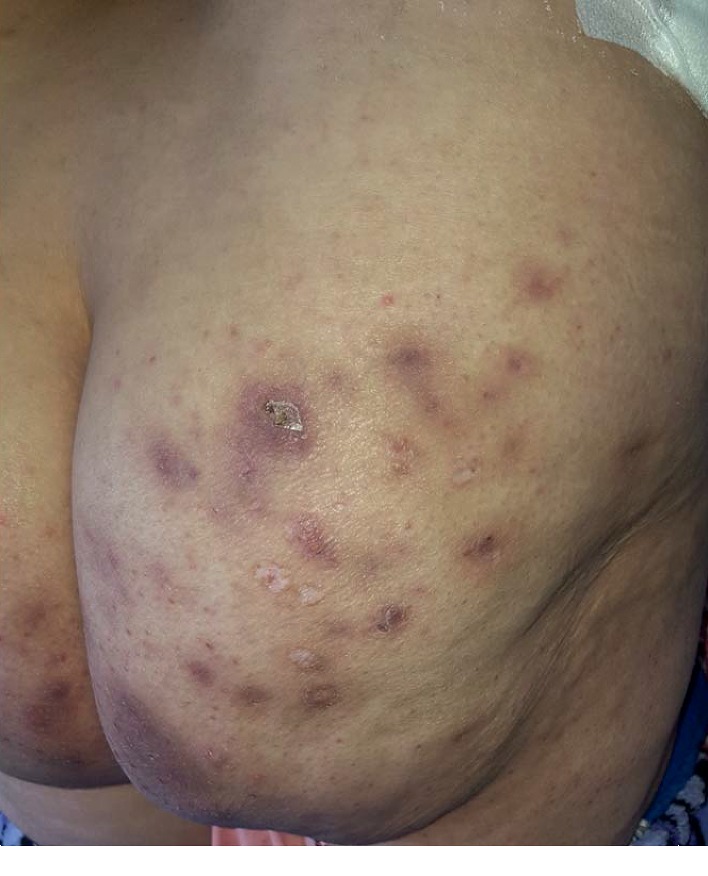
HS stage I of Hurley, with gluteal predominance.

The patient attended our dermatology department with an HS stage I of Hurley, with gluteal predominance (Figure 1). She had been treated initially with isotretinoin 0.5 mg/day, along with topical benzoyl peroxide and hygienic measures upon 3 months, which had offered minimal relief. It was at this stage the chronology between leflunomide introduction and the flare of HS raised the possibility of a leflunomide-induced HS. Hence, the medication was substituted by methotrexate and rituximab. Major improvement in inflammatory nodules was observed in a few weeks after withdrawal of leflunomide. Although methotrexate and rituximab are not effective for the treatment of HS, the role of these drugs can not be ruled out. The Naranjo score was 4, corresponding to a possible reaction.

Remission was maintained at 18 months follow-up visit.

### 2.2. Case 2

**Figure 2:**
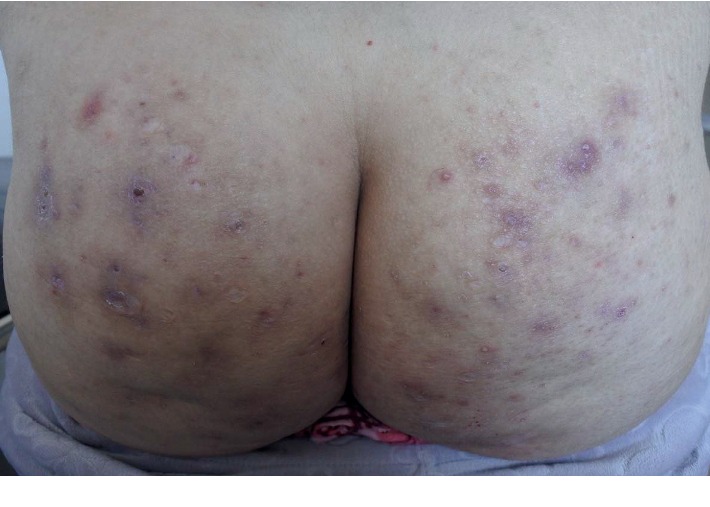
Cicatricial lesions on the buttocks after discontinuation of leflunomide.

A 34-year-old woman, nonsmoker, with a BMI of 29 kg/m^2^ and no relevant family history of HS, and on follow-up for a rheumatoid arthritis on treatment with prednisone 5 mg/day and leflunomide 20 mg/day for 6 years, was referred to our outpatient clinic with a 3-year history of suppurative nodules of the buttocks. Physical examination at the first consultation revealed only cicatricial lesions (Figure 2). The patient noted a surprising improvement the few weeks before the medical appointment. History showed a withdrawal of leflunomide for inventory shortage 1 month before. The patient was moved to methotrexate, and no novel nodules were seen. The Naranjo score for this patient was 5, corresponding to a probable reaction.

## 3. Discussion

Drug-induced HS is rare. Frew et al. identified 48 individual cases in a recent review of the literature, with none concerning leflunomide [2]. Anti-TNF drugs are the most implicated, and an important finding is that all the medications reported modulate aspects of the innate immune system, specifically Toll-like receptor (TLR) activity.

Leflunomide is an immunomodulatory agent used in rheumatoid arthritis that inhibits de-novo pyrimidine synthesis and therefore suppresses ADN expression, leading to a decrease in T cell and B cell [3]. Its action on modulation of TLR activity is well recognized [4].

Inflammatory rheumatism, specially spondyloarthropathy, is thought to be frequently associated with HS [5]. Nevertheless, the chronology of flares of HS in relation to leflunomide onset, as well as the improvement after discontinuation of the drug, strengthens the possibility of drug-associated etiology.

The association of overweight and rheumatoid arthritis may have precipitated the onset of HS by the suggested drug.

Finally, the incubation period reported in the literature ranges from 1 to 120 months, with an average of 20.5 months [2]. In the 2 cases reported above, incubation periods were of 36 and 3 months, respectively.

## 4. Conclusion

Taken together, these data suggest that leflunomide is a suspect of induction of HS. Further publications may affirm or deny this link.
